# Enhancing Radiation Shielding Capabilities with Epoxy-Resin Composites Reinforced with Coral-Derived Calcium Carbonate Fillers

**DOI:** 10.3390/polym17010113

**Published:** 2025-01-04

**Authors:** Gunjanaporn Tochaikul, Nuttapol Tanadchangsaeng, Anuchan Panaksri, Nutthapong Moonkum

**Affiliations:** 1 Faculty of Radiological Technology, Rangsit University, Pathumthani 12000, Thailand; gunjanaporn.t@rsu.ac.th; 2 College of Biomedical Engineering, Rangsit University, Pathumthani 12000, Thailand; nuttapol.t@rsu.ac.th (N.T.); anuchan.p@rsu.ac.th (A.P.)

**Keywords:** epoxy–resin composites, coral-derived calcium carbonate, radiation shielding, mechanical properties, sustainable materials

## Abstract

This study investigates the development of epoxy–resin composites reinforced with coral-derived calcium carbonate (CaCO_3_) fillers for enhanced radiation shielding and mechanical properties. Leveraging the high calcium content and density of coral, composites were prepared with filler weight fractions of 0%, 25%, and 50%. SEM and EDS analyses revealed that higher filler concentrations (50%) increased particle agglomeration, affecting matrix uniformity. Mechanical testing showed that while the tensile and flexural strengths decreased with the increased filler content, the compressive strength significantly improved, reaching 135 MPa at a 50% coral content. Radiation shielding evaluations demonstrated enhanced attenuation with a higher filler content, achieving 39.63% absorption at 60 kVp for the 50% coral composite. However, the shielding efficiency was notably lower compared to lead, which achieves over 99% absorption at similar energy levels. These quantitative comparisons highlight the material’s limitations in high-radiation environments but emphasize its suitability for moderate shielding applications. Despite their lower shielding efficiency, the composites provide an environmentally friendly and non-toxic alternative to lead, aligning with sustainability goals. Future work should focus on optimizing filler dispersion, mitigating agglomeration, and exploring hybrid systems to enhance the shielding efficiency and mechanical properties. The further quantitative evaluation of parameters such as Zeff and cross-sections is recommended to comprehensively assess the material’s performance.

## 1. Introduction

Radiation shielding materials play a vital role across numerous fields, such as medical imaging and nuclear energy [1], by reducing radiation exposure and protecting human health [2]. Traditionally, materials with high atomic numbers, such as lead (Pb), have been widely used for radiation shielding due to their excellent attenuation properties [3]. Lead, owing to its high atomic number and density, efficiently absorbs ionizing radiation, making it a common choice in shielding applications [4]. However, its toxicity and adverse environmental impact have raised concerns, necessitating the development of safer, more sustainable alternatives [5].

In radiation shielding research, various fillers like barium sulfate (BaSO_4_) [6], tungsten trioxide (WO_3_) [7], gadolinium oxide (Gd_2_O_3_) [8] and bismuth oxide (Bi_2_O_3_) [9] are frequently incorporated into polymer matrices due to their effective radiation attenuation properties. Studies indicate that silicone rubber with 70% BaSO_4_ and Bi_2_O_3_ achieves a notable 90.19–97.48% radiation absorption at 120 kVp, offering a flexible, eco-friendly alternative for reducing diagnostic X-ray exposure [10]. Additionally, flexible thermoplastic composites with tungsten–bismuth (W/Bi) and bismuth tungsten oxide fillers (20–100% ratios) have shown strong attenuation at X-ray energies of 80–120 kVp and with gamma rays ranging from 59 to 960 keV, with W/Bi proving particularly effective due to its superior attenuation coefficients and flexibility—ideal for medical radiation protection [11]. In glass-based shielding, Gd_2_O_3_-doped samples (0–10 mol%) were evaluated for gamma (0.015–15 MeV) and neutron (0.5–10 MeV) shielding. The sample with 10 mol% Gd_2_O_3_ (GSNBC4) showed the highest gamma attenuation, while a higher B_2_O_3_ content (54.5 mol%) in GSNBC1 yielded the greatest neutron removal effectiveness [12]. For natural rubber (NR) composites, gamma shielding comparisons between lead oxide (PbO) and Bi_2_O_3_ fillers revealed that NR/PbO composites offer enhanced tensile strength, while NR/Bi_2_O_3_ composites provide better oil resistance and thermal stability. Both types demonstrated excellent gamma shielding, surpassing concrete and hematite-serpentine [13].

In recent years, researchers have sought alternatives that integrate non-toxic or eco-friendly materials [14]. One promising strategy involves harnessing high-atomic-number elements found in natural waste products [15]. The combination of these high-atomic-number elements, their abundant availability, and low cost in certain natural wastes presents an innovative path toward developing sustainable radiation shielding materials [16]. For example, composites derived from eggshells have demonstrated promise due to their high calcium content [17], which, when ground into powder and mixed with a binder, provides shielding properties comparable to conventional materials [18]. Similarly, waste-based materials such as rice husk ash and seashell composites are being explored for their effectiveness in radiation shielding applications that demand low-to-moderate protection levels [19,20]. “Beach debris” or “marine debris” includes human-made waste in coastal areas, such as plastics [21], metals, glass [22] and, notably, coral fragments [23]. Coral fragments, often remnants of coral reefs, can be affected by ocean acidification, warming, and physical damage [24], but they also offer potential for innovative applications, like natural filtration [25] or even sustainable materials for scientific research [26]. Rich in calcium carbonate, coral debris has potential uses in construction [27], water treatment [28] and pharmaceuticals [29]. Scientific research on coral debris provides insights into reef health and the effects of pollution, underscoring the need for effective waste management to protect marine environments [30]. Integrating coral debris into broader environmental strategies highlights its value in conservation and pollution mitigation efforts [31].

This study investigates the development of epoxy–resin composites reinforced with coral-derived calcium carbonate fillers as a sustainable alternative to lead-based radiation shielding materials. Current eco-friendly solutions often fail to balance mechanical strength and radiation attenuation efficiency, highlighting the need for innovative materials. This research addresses these gaps by leveraging the high-density and structural properties of calcium carbonate derived from coral, aiming to create an effective composite for X-ray protection. The study examines the mechanical and shielding properties of composites with varying filler concentrations, focusing on their applicability in medical, industrial, and nuclear settings. The novelty lies in utilizing naturally sourced coral fillers to enhance material performance while promoting environmental sustainability. Limitations include their reduced tensile and flexural strength at higher filler concentrations and lower shielding efficiency compared to lead. Future directions include optimizing filler dispersion, exploring hybrid systems, and conducting durability testing to broaden the composites’ potential applications.

## 2. Materials and Methods

### 2.1. Raw Material and Coral Processing

The epoxy resin used in this study was obtained from Resin Lab, Thailand. The resin consisted of diglycidyl ether of bisphenol A (DGEBA), a commonly used epoxy monomer due to its excellent mechanical properties, thermal stability, and adhesive characteristics [32]. The coral fragments used in this study were sourced from a certified domestic supplier in Thailand (Lam Luk Ka Khlong 4 Market, Pathumthani, Thailand), ensuring compliance with environmental conservation laws and regulations. These fragments were verified to originate from naturally degraded coral, without harvesting from ecosystems that could be adversely affected.

**Figure 1:**
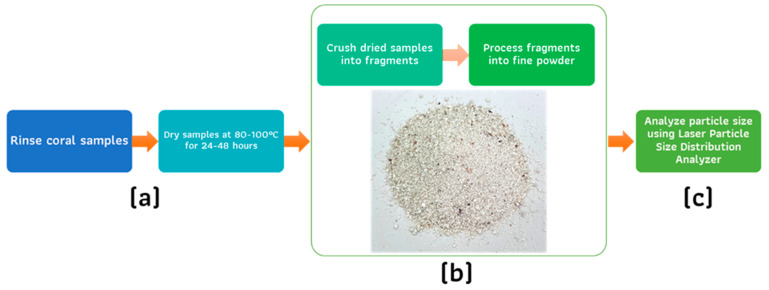
Preparation and analysis of coral samples: (**a**) clean and dry samples, (**b**) fragment and pulverize into fine powder, (**c**) analyze particle size using laser-based distribution techniques.

Figure 1 illustrates the preparation process for the coral samples. Initially, the coral samples were rinsed thoroughly with seawater to eliminate loose debris and marine organisms, ensuring a clean and uncontaminated starting material. Following this, the cleaned samples were dried in an oven at a controlled temperature range of 80–100 °C for 24–48 h to ensure the complete removal of moisture. Once dried, the coral samples were mechanically crushed into smaller fragments using a crusher. These fragments were subsequently ground into a fine powder using a ball mill and pestle to achieve a uniform particle size. The particle size distribution (PSD) of the resulting coral powder was then analyzed using a Laser Particle Size Distribution Analyzer. This analysis provided precise data on the size range and uniformity of the particles, confirming their suitability for use in composite material applications.

### 2.2. Composite Material Preparation Involves Blending Coral Powder with Epoxy Resin

**Figure 2:**
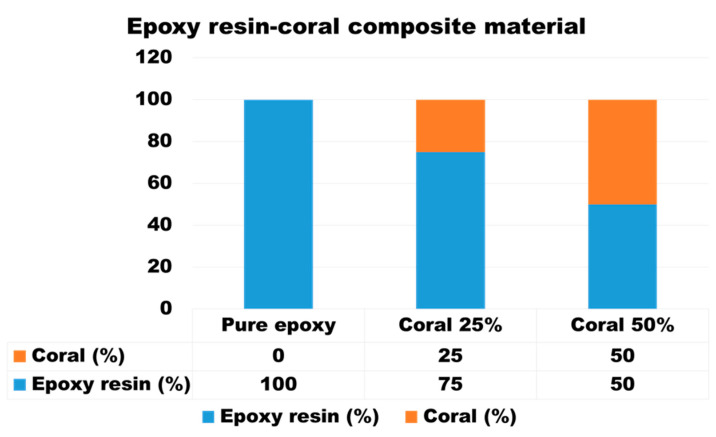
Composition distribution of epoxy resin, hardener, and coral powder at 0%, 25%, and 50% weight fractions.

The preparation of the epoxy–resin matrix involved combining the epoxy resin with the hardener in accordance with the manufacturer’s specified guidelines. The required amounts of epoxy resin and hardener were precisely measured using a calibrated scale, adhering to the recommended 2:1 ratio by weight or volume. The necessary quantities of coral powder to achieve weight fractions of 0%, 25%, and 50% were calculated relative to the composite’s total weight, as illustrated in Figure 2.

The selection of these specific weight fractions (0%, 25%, and 50%) was based on their relevance in balancing mechanical properties and their radiation shielding performance. These compositions allow for the systematic evaluation of the effect of the filler concentration on composite characteristics, such as tensile and compressive strength, as well as the radiation attenuation efficiency. This range provides insights into the trade-offs between mechanical performance and shielding capability, helping to identify the optimal filler content for various applications.

**Figure 3:**
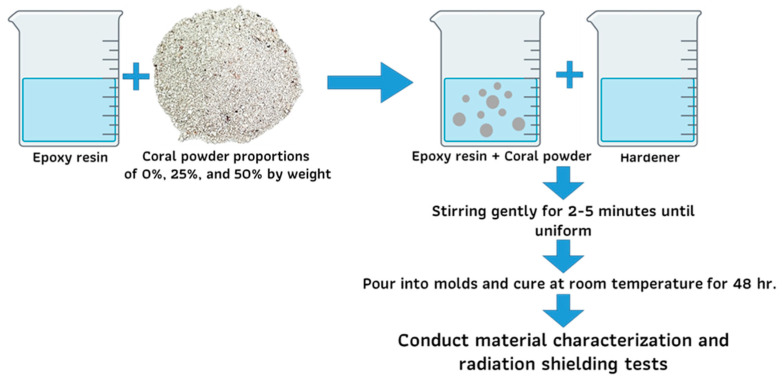
Process of preparing epoxy–resin composite samples with calcium carbonate fillers.

In a clean and contaminant-free container, the epoxy resin and hardener were mixed thoroughly by gentle stirring for 2–5 min until a uniform mixture was achieved. The pre-calculated coral powder was then gradually added to the mixture while using a mechanical stirrer to ensure homogeneity and prevent the formation of agglomerates. The mixing process continued for an additional 5 min to achieve the consistent distribution of the coral powder within the matrix. The prepared mixture was subsequently poured into molds and allowed to cure at room temperature for a duration of 48 h. After curing, the samples were ready for further material characterization and radiation shielding performance evaluation, as detailed in Figure 3.

### 2.3. Comprehensive Characterization of Epoxy-Resin Composites Reinforced with Coral-Derived Calcium Carbonate

A comprehensive characterization was conducted to evaluate the quality and performance of epoxy–resin composites reinforced with coral-derived calcium carbonate fillers. The analysis included multiple techniques: X-ray Fluorescence (XRF) for assessing the elemental composition, Particle Size Distribution (PSD) analysis for assessing particle uniformity, Scanning Electron Microscopy (SEM) for examining the surface morphology and microstructural features, and mechanical testing to determine the composites’ tensile, compressive, and flexural properties.

The raw materials utilized in this study are illustrated in Figure 1b. The coral sample was analyzed for its elemental composition using Wavelength Dispersive X-ray Fluorescence (XRF) spectrometry with a Bruker S8 Tiger XRF spectrometer. The sample, prepared in powdered form, underwent analysis, with the elemental composition being determined using theoretical formulas and fundamental parameter calculations, with results expressed as oxide concentrations.

The particle size distribution (PSD) of the coral powder was assessed using a Laser Particle Size Distribution Analyzer (Model LA960V2). The sample was dispersed in a liquid medium to ensure proper suspension and minimize agglomeration. The analysis utilized refractive indices of 1.658 for calcium carbonate and 1.333 for water. The instrument parameters included a circulation speed of 5 and an agitation speed of 7, with ultrasound disabled. Transmittance values of 89.4% (red channel) and 92.5% (blue channel) were recorded, ensuring the reliability of the optical measurements.

Morphological analysis was conducted using a JEOL JSM-IT300 scanning electron microscope (USA) operating at 15 kV. Samples measuring 1.0 × 1.0 cm^2^ were sputter-coated with a thin layer of gold for enhanced imaging quality. Micrographs at magnifications of 500× and 2000× provided a detailed visualization of the surface morphology and microstructural features. Energy-Dispersive X-Ray Spectroscopy (EDS), integrated with SEM, enabled elemental mapping to confirm the uniform distribution and concentration of calcium carbonate within the epoxy matrix.

Mechanical testing included the assessment of tensile strength, compressive strength, and flexural strength, which are critical parameters for evaluating the structural performance of composites. Tensile testing was conducted using a TK-10TX tensile testing machine (BEMACS, Osaka, Japan) with a load capacity of 1 to 10 tons. For tensile strength, the samples were subjected to incremental tensile forces until failure, and the ultimate tensile strength was recorded in megapascals (MPa). The compressive strength was evaluated by applying compressive loads to measure the material’s ability to withstand axial forces, while the flexural strength was determined by subjecting the samples to three-point bending tests to measure their resistance to deformation under bending loads. These combined measurements provided a comprehensive evaluation of the mechanical properties, highlighting the balance between strength and flexibility in the composite materials.

The theoretical calculation of mechanical properties, including tensile strength, compressive strength, and flexural strength, is critical for evaluating the performance of epoxy–resin composites reinforced with coral-derived calcium carbonate fillers. The tensile strength ($\sigma_{ts}$) is determined using the formula

(1) $$\sigma_{ts}=\frac{F_{t}}{A}$$

where $F_{t}$ is the maximum tensile force applied before failure (in Newtons), and *A* is the cross-sectional area of the specimen (in square meters). This calculation quantifies the material’s resistance to uniaxial tensile loads.

Compressive strength ($\sigma_{c}$) is calculated as

(2) $$\sigma_{c}=\frac{F_{c}}{A}$$

where $F_{c}$ represents the maximum compressive force before failure, and *A* is the cross-sectional area. This formula evaluates the material’s capacity to withstand compressive loads without deformation.

Flexural strength ($\sigma_{f}$), a measure of the material’s resistance to bending, is derived using the three-point bending test formula

(3) $$\sigma_{f}=\frac{3FL}{2bd^{2}}$$

where *F* is the applied load at the midpoint, *L* is the span length, *b* is the specimen’s width, and *d* is its thickness.

### 2.4. Experimental Setup and Evaluation of Radiation Shielding Effectiveness

**Figure 4:**
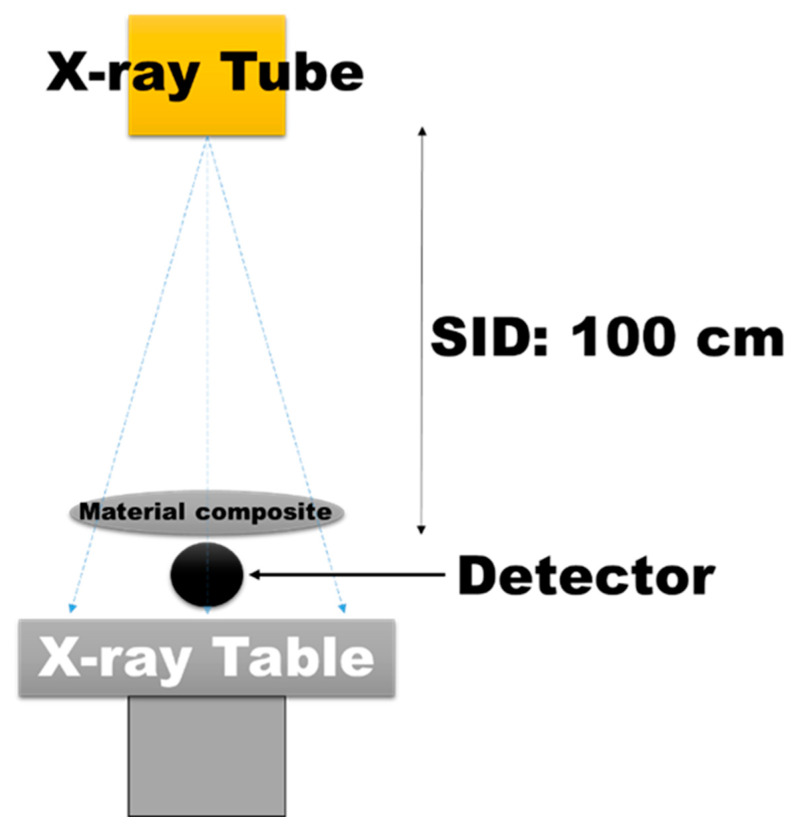
Experimental setup for radiation attenuation measurements using a medical X-ray diagnostic unit and Radcal accuGold detector.

The experimental setup involved the use of a medical X-ray diagnostic unit (RADspeed Fit, Shimadzu Corporation, Kyoto, Japan) configured to operate at exposure parameters of 60, 80, 100, and 120 kVp with a fixed tube current of 5 mAs. The source-to-image distance (SID) was maintained at a constant 100 cm throughout the experiment. A Radcal AccuGold detector was positioned to fully encompass the X-ray beam output, ensuring complete coverage of the field size. Prior to conducting the tests, both the X-ray unit and the radiation detector were calibrated according to the manufacturers’ guidelines to ensure accurate and reliable measurements, as illustrated in Figure 4.

To evaluate the shielding properties of the tested materials, the percentage absorption of radiation dose (%Absorption) was calculated using the following formula:

(4) $$\%\mathrm{Absorption}=(\frac{D_{baseline}-D_{shielded}}{D_{baseline}})\times 100$$

where *D_baseline_* is the radiation dose measured without shielding, and *D_shielded_* is the radiation dose measured with the shielding material [33].

The linear attenuation coefficient (*μ*), representing the material’s capacity to attenuate X-rays, was calculated using the exponential attenuation law:

(5) $$I=I_{0}e^{-\mu x}$$

where *I* is the transmitted intensity, *I_0_* is the initial intensity and *x* is the thickness of the shielding material [10]. By rearranging the equation, *μ* can be derived as follows:

(6) $$\mu =\frac{1}{x}\ln\frac{I_{o}}{I}$$

This relationship enables the calculation of *μ* based on experimentally measured values of *I* and *I_0_*, along with the known material thickness x. The linear attenuation coefficient serves as a critical parameter for assessing the material’s X-ray shielding efficiency, with higher *μ* values indicating superior attenuation capabilities.

Additionally, the Half-Value Layer (HVL), which signifies the thickness of material required to reduce the X-ray intensity by half, was determined using the following equation [34]:

(7) $$\mathrm{HVL}=\frac{ln(2)}{\mu }$$

The parameters calculated, including the percentage absorption (%Absorption), linear attenuation coefficient (*μ*), and HVL, provided a comprehensive assessment of the shielding effectiveness of the tested materials. These metrics offer valuable insights into the material’s potential for radiation protection applications.

To comprehensively evaluate the radiation shielding performance of epoxy–resin composites reinforced with coral-derived calcium carbonate fillers, additional parameters, including the atomic cross-section ($\sigma_{t}$), electronic cross-section ($\sigma_{e}$) and effective atomic number ($Z_{eff}$), are strongly recommended. These parameters provide critical insights into the mechanisms implicated in the interactions between photons and the material, enabling a more thorough assessment of its shielding capabilities.

The atomic cross-section ($\sigma_{t}$), which quantifies the probability of photon interactions per atom, is calculated using the formula

(8) $$\sigma_{t}=\frac{\mu }{N_{a}}$$

where *μ* is the linear attenuation coefficient (cm^−1^) and $N_{a}$ is the number of atoms per unit volume.

The value of $N_{a}$ is determined by

(9) $$N_{a}=\frac{{\rho N}_{A}}{A}$$

where ρ represents the material density (g/cm^3^), $N_{A}$ is Avogadro’s number, and *A* is the atomic weight.

The electronic cross-section ($\sigma_{e}$) reflects the probability of photon interactions per electron and is calculated as

(10) $$\sigma_{e}=\frac{\sigma_{t}}{Z_{eff}}$$

where $Z_{eff}$ is the effective atomic number of the material.

The effective atomic number ($Z_{eff}$) itself is an essential parameter for understanding photon interactions, particularly in composite materials. It is determined using the formula

(11) $$Z_{eff}={\left(\frac{\sum \omega_{i}Z_{i}^{p}}{\sum \omega_{i}A_{i}^{p}}\right)}^{1/p}$$

where $\omega_{i}$ is the weight fraction of the *i*-th element, $Z_{i}$ and $A_{i}$ are the atomic number and atomic weight, respectively, and *p* is a parameter dependent on the photon energy range, typically set to 3–4 for intermediate energies.

## 3. Results

### 3.1. Elemental Composition of Coral Sample Analyzed by X-Ray Fluorescence

The analysis of the coral sample revealed a high calcium oxide (CaO) content, accounting for 51.60% by weight, which confirms the predominance of calcium as the primary component. This indicates the suitability of the coral material for applications requiring a high calcium content, such as filler materials in composites. In addition to calcium oxide, the analysis detected magnesium oxide (MgO) at 1.06%, contributing to the structural properties of the material. Silicon dioxide (SiO_2_) was detected at 0.66%, indicating the presence of trace silicate impurities that may influence the mechanical or chemical properties of the material. Other minor constituents included potassium oxide (K_2_O) and sodium oxide (Na_2_O), present in trace amounts in Table 1. The comprehensive elemental profile underscores the potential use of the coral-derived material in applications that leverage its calcium-rich composition while also considering the impact of minor elements on the overall material performance.

### 3.2. Particle Size Distribution (PSD) Analysis of Coral Powder

**Figure 5:**
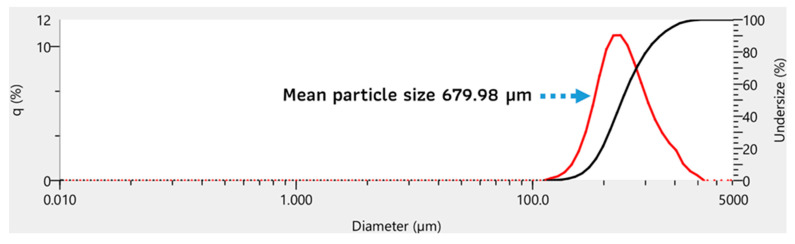
Particle size distribution (PSD) of coral powder showing mean particle size of 679.98 µm.

The particle size distribution (PSD) analysis of the coral powder sample provided detailed insights into its particle size characteristics. The D(v,0.1) value, representing the size below which 10% of the particles are distributed, was 315.75 µm. The median particle size, or D(v,0.5), was measured at 572.17 µm, indicating that half of the sample volume consists of particles smaller than this size. The D(v,0.9) value, which accounts for 90% of the particles, was recorded at 1206.94 µm. The mean particle size of the sample was calculated as 679.98 µm, reflecting the average size across the distribution in Figure 5.

These results indicate a broad range of particle sizes, with the majority falling within the micrometer scale. The data demonstrate that the coral powder is well prepared and suitable for applications where a consistent particle size distribution is essential, such as in composite materials. The findings also confirm the effectiveness of the preparation process in ensuring uniformity and reducing particle agglomeration.

### 3.3. Morphological Characterization of Epoxy–Resin Composites Incorporating Coral Powder at Varying Weight Fractions

**Figure 6:**
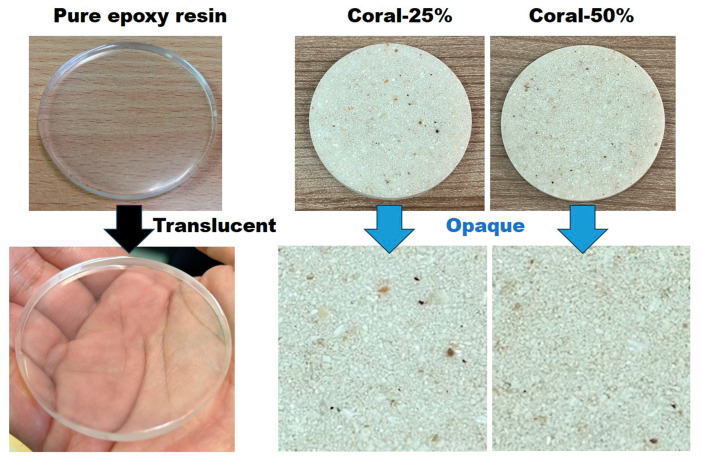
Morphological appearance of epoxy–resin composites with 0%, 25% and 50% coral powder weight fractions.

The prepared epoxy–resin matrix was evaluated after incorporating coral powder at varying weight fractions of 0%, 25%, and 50%. As shown in Figure 6, the matrix without coral powder (0% weight fraction) exhibits a clear and transparent appearance, indicative of pure resin properties. At a 25% weight fraction of coral powder, the composite transitions to a slightly opaque texture, with visible fine particles uniformly dispersed throughout the matrix. When the coral powder content increases to 50%, the composite becomes fully opaque, with a dense distribution of coral particles, demonstrating significant filler integration.

These observations highlight the progressive impact of an increasing coral powder content on the composite’s visual and structural properties. The uniform dispersion of coral powder in the resin indicates successful mixing and integration, confirming adherence to the preparation methodology. The results provide critical insights into the composite’s morphological changes due to varying filler concentrations, which are essential for subsequent mechanical and functional evaluations.

### 3.4. Physical Characteristics of Coral Powder SAMPLE Analyzed via SEM-EDS

**Figure 7:**
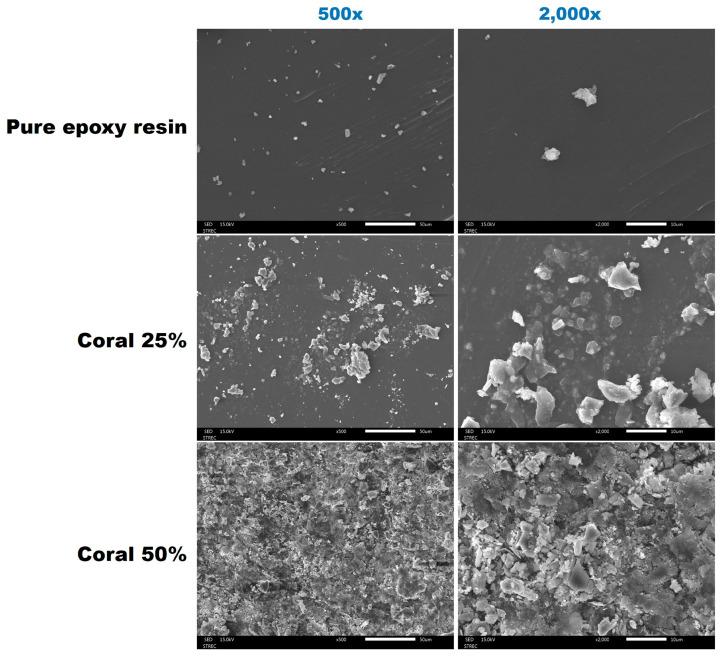
SEM images of epoxy–resin composites incorporating coral powder at weight fractions of 0%, 25%, and 50%, showing the surface morphology at different magnifications.

The physical characteristics of coral powder incorporated into an epoxy–resin matrix at varying weight fractions (0%, 25%, and 50%) were evaluated using photographic and SEM-EDS analyses, as shown in Figure 7. The results revealed significant changes in the surface morphology and elemental composition as the coral powder content increased.

SEM imaging demonstrated notable variations in surface morphology across the samples. The control sample (0% coral powder) exhibited a smooth, homogeneous texture, indicative of pure epoxy resin. At 25% coral powder, dispersed coral particles were embedded within the matrix, forming localized clusters. The particles appeared irregular in shape, reflecting the granular nature of the coral material. In contrast, the 50% coral powder sample displayed a more heterogeneous structure, with densely packed particles, an increased surface roughness, and evident agglomeration. These observations suggest that a higher filler content disrupts the continuity of the resin matrix, potentially impacting the composite’s mechanical properties.

The addition of coral powder significantly altered the physical characteristics of the epoxy–resin matrix. While the 25% coral powder sample achieved a relatively uniform dispersion and enhanced surface roughness, the 50% sample suffered from particle agglomeration, void formation, and reduced matrix homogeneity. These findings highlight the potential use of coral powder as a filler material and underscore the need to optimize the filler content to balance structural integrity and material performance.

**Figure 8:**
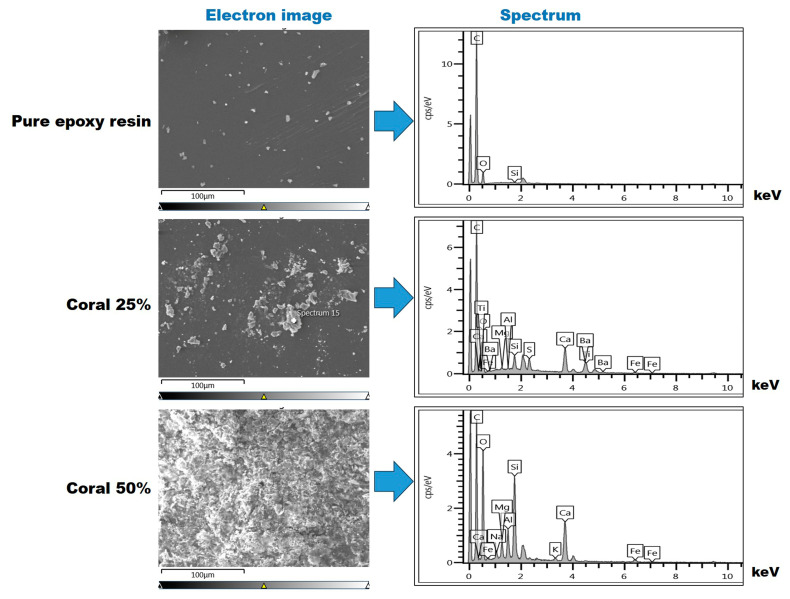
SEM-EDS analysis showing electron images and elemental spectra of epoxy composites with 0%, 25%, and 50% coral powder.

The Energy-Dispersive X-ray Spectroscopy (EDS) analysis of the epoxy–resin matrix and coral composites with coral powder weight fractions of 0%, 25%, and 50%, as presented in Figure 8, provided significant insights into the elemental distribution and compositional changes with a varying filler content.

For the pure epoxy sample (0% coral powder), the elemental composition primarily consisted of carbon (C) and oxygen (O), with weight percentages of 81.24% and 18.55%, respectively, as shown in Table 2. This distribution reflects the organic and polymeric structure of the epoxy resin. Trace amounts of silicon (Si) were detected at 0.21%, likely originating from additives or minor contaminants in the resin formulation.

In the 25% coral powder composite, a marked reduction in the carbon content was observed (52.34%), along with an increase in the oxygen content (23.18%). The addition of coral powder introduced new elements such as calcium (Ca, 7.55%), barium (Ba, 8.58%), and titanium (Ti, 2.81%), which are consistent with the mineralogical composition of coral. Additionally, minor elements including sulfur (S, 2.2%), magnesium (Mg, 0.26%), and aluminum (Al, 0.16%) were identified. These results confirm the successful incorporation of coral particles into the resin matrix.

In the 50% coral powder composite, further shifts in the elemental composition were observed. The carbon content decreased to 46.69%, reflecting the increased dilution of the epoxy matrix due to the higher filler fraction. The oxygen levels rose to 31.79%, indicative of the oxygen-rich nature of coral. The calcium content slightly increased to 8.28%, while barium was no longer detected. The silicon (Si, 6.91%) and magnesium (Mg, 1.4%) concentrations were significantly higher compared to the 25% composite, suggesting the increased contribution of silica-based components from the coral powder. Potassium (K, 0.43%) and sodium (Na, 0.7%) were also newly detected, aligning with the mineral composition of coral and its interaction with the resin matrix.

The EDS findings are consistent with the SEM observations, which revealed increasing coral particle agglomeration at higher filler contents. The greater diversity and concentration of elements such as Ca, Si, and Mg further validate the embedding of coral powder into the matrix and the associated shift in its physical and chemical properties. This analysis underscores the compositional transition from a predominantly polymeric epoxy matrix in the control sample to a mineral-enriched composite as the coral powder content increases. These findings highlight the potential of coral powder as a functional filler in composite materials, particularly for applications requiring specific elemental profiles.

### 3.5. Radiation Attenuation Properties of Epoxy Composites Reinforced with Coral-Derived Calcium Carbonate

**Figure 9:**
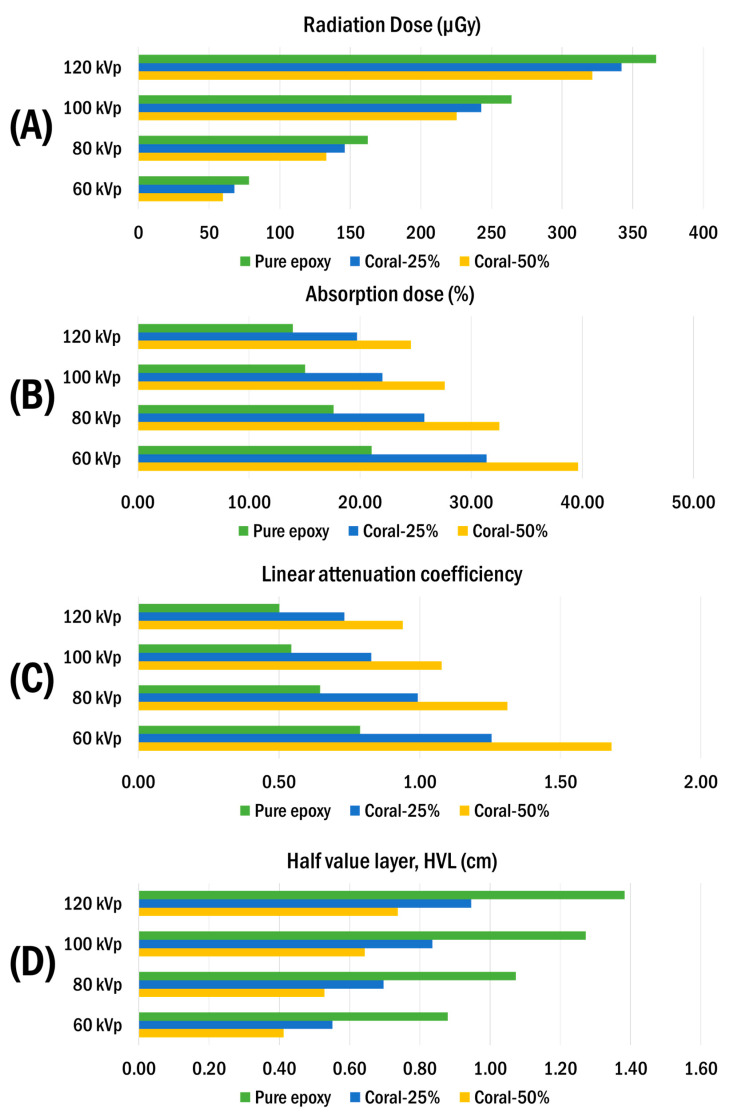
(**A**) Radiation attenuation performance of epoxy composites with coral fillers at 0%, 25%, and 50%: (**B**) %absorption of radiation dose, (**C**) linear attenuation coefficient (µ) and (**D**) half-value layer (HVL).

The radiation attenuation properties of epoxy composites reinforced with coral-derived calcium carbonate were assessed at varying filler concentrations (0%, 25%, and 50%) under different X-ray energy levels (60 kVp, 80 kVp, 100 kVp, and 120 kVp). The results, presented in Figure 9, demonstrate a marked improvement in the attenuation performance with an increasing coral filler content.

For the radiation dose measurements, the pure epoxy sample recorded the highest exposure values, ranging from 78.21 µGy at 60 kVp to 366.7 µGy at 120 kVp. In contrast, the composites containing 25% and 50% coral filler exhibited significantly reduced radiation doses. Notably, the 50% coral composite recorded doses as low as 59.81 µGy at 60 kVp and 321.5 µGy at 120 kVp (Figure 9A).

The absorption dose analysis followed a similar trend, with the percentage absorption increasing with the coral filler content. At 60 kVp, the pure epoxy sample achieved an absorption rate of 21.06%, while the 25% and 50% coral composites demonstrated improved absorption rates of 31.40% and 39.63%, respectively (Figure 9B).

The linear attenuation coefficient values corroborated these findings, revealing an enhanced attenuation performance with a higher coral filler content. The pure epoxy sample exhibited the lowest coefficient (e.g., 0.79 cm^−1^ at 60 kVp), whereas the 25% and 50% coral composites showed significantly higher coefficients of 1.26 cm^−1^ and 1.68 cm^−1^, respectively (Figure 9C).

The half-value layer (HVL) measurements further highlighted the impact of coral filler addition on the shielding performance. The HVL of pure epoxy was 0.88 cm at 60 kVp, which was reduced to 0.55 cm and 0.41 cm for the 25% and 50% coral composites, respectively (Figure 9D). These reductions in HVL values reflect the improved attenuation efficiency of the coral-reinforced composites. In conclusion, the incorporation of coral-derived calcium carbonate into epoxy composites significantly enhances their radiation shielding properties. The results indicate that increasing the coral filler content improves the attenuation performance, absorption capacity, and linear attenuation coefficients while reducing the HVL, making these composites promising candidates for radiation protection applications.

### 3.6. Comparison of Radiation Shielding Properties Between High Filler Coral-Derived Calcium Carbonate Composite and Lead

The radiation shielding performance of a high-filler composite containing 50% coral-derived calcium carbonate was evaluated in comparison to a standard lead apron (0.5 mm Pb equivalent, RayShield^®^ USA) under 120 kV X-ray energy. The comparison focused on key parameters, including the radiation dose, absorption efficiency, linear attenuation coefficient, and half-value layer (HVL), as summarized in Table 3.

The lead apron exhibited a superior shielding performance across all measured metrics. The radiation dose transmitted through the lead apron was 27.75 µGy, significantly lower than the 321.5 µGy recorded for the coral-derived composite. This substantial difference underscores the higher attenuation capacity of lead, achieving an absorption efficiency of 93.49%, compared to 24.57% for the coral-based composite.

The linear attenuation coefficient of lead was determined to be 5.46 cm^−1^, markedly higher than the 0.94 cm^−1^ observed for the coral composite. This higher coefficient reflects the exceptional capacity of lead to reduce the X-ray intensity over a shorter path length. Consistent with this, the half-value layer (HVL) for lead was measured at 0.13 cm, being significantly smaller than the 0.74 cm required for the coral composite. The lower HVL of lead highlights its superior density and atomic number, which contribute to its enhanced attenuation properties.

In conclusion, the lead apron demonstrated a significantly greater radiation shielding efficiency compared to the coral-derived composite under 120 kV X-ray exposure. While the coral composite shows promise as an environmentally friendly, lead-free alternative, its current shielding performance is markedly inferior to that of conventional lead-based materials. Future efforts to optimize the formulation and structure of the coral composite could further improve its attenuation capabilities, potentially making it a viable option for specific applications requiring sustainable shielding materials.

### 3.7. Mechanical Properties for Epoxy-Resin COMPOSITES Incorporating Coral-Derived Calcium Carbonate

**Figure 10:**
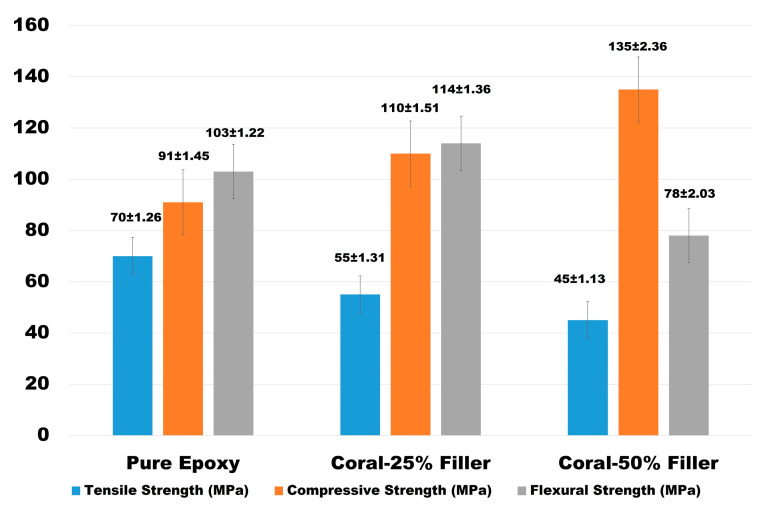
Mechanical properties of epoxy composites with varying coral-derived calcium carbonate filler contents.

The mechanical properties of epoxy composites reinforced with varying weight fractions of coral-derived calcium carbonate fillers (0%, 25%, and 50%) were assessed in terms of tensile strength, compressive strength, and flexural strength. The findings, presented in Figure 10**,** illustrate the impact of filler content on the mechanical behavior of the composites.

For tensile strength, the pure epoxy sample exhibited the highest value at 70 MPa, reflecting the cohesive and uninterrupted polymeric structure of the matrix. The addition of 25% coral filler reduced the tensile strength to 55 MPa, while increasing the filler content to 50% further decreased this property to 45 MPa. These reductions are attributed to the increased brittleness and stress concentrations introduced by the dispersed filler particles within the matrix.

In terms of compressive strength, the pure epoxy sample showed a baseline value of 91 MPa. The incorporation of 25% coral filler enhanced this property to 110 MPa, and further addition to 50% filler increased the compressive strength to 135 MPa. This improvement can be attributed to the high-density coral filler particles, which reinforce the composite under compressive loads, increasing its load-bearing capacity.

For flexural strength, the pure epoxy sample achieved a value of 103 MPa, indicating its resistance to bending forces. The addition of 25% coral filler slightly improved the flexural strength to 114 MPa, likely due to the increased rigidity provided by the filler. However, at 50% filler content, the flexural strength decreased significantly to 78 MPa, reflecting a reduction in matrix flexibility and the increased brittleness associated with higher filler concentrations.

These results highlight the trade-offs inherent in increasing coral-derived filler content. While compressive strength improves significantly with higher filler percentages, tensile and flexural properties decline due to the brittleness and stress concentration effects of the fillers. These findings emphasize the importance of optimizing filler content to achieve a balance between mechanical performance and application-specific requirements.

## 4. Discussion

The findings of this study emphasize the significant benefits of utilizing coral-derived calcium carbonate as a functional material in composite applications. The high calcium oxide (CaO) content, measured at 51.60% by weight, underscores its potential as a structural filler. Calcium-rich fillers are well documented for their ability to enhance the mechanical properties of composites, particularly in improving the compressive strength and tensile performance, making them ideal for applications such as radiation shielding, construction, and biomedical devices [35]. In terms of mechanical properties, the incorporation of coral-derived calcium carbonate significantly enhances the compressive strength of epoxy composites, a critical attribute for applications requiring materials capable of withstanding high compressive loads, such as structural panels or protective coatings. However, achieving a balance between the filler content and flexibility is essential to maintain optimal performance, as observed in related studies on bio-based composite materials [36]. The addition of calcium carbonate (CaCO_3_) fillers influences the mechanical properties, including the tensile, compressive, and flexural strength [37]. While the tensile strength decreases due to stress concentration points and potential weak interfacial bonding, the compressive strength improves due to the rigidity of the CaCO_3_ particles. Flexural strength, on the other hand, shows a complex relationship, improving at lower filler contents but decreasing at higher concentrations due to increased brittleness [38,39,40].

The use of coral-derived calcium carbonate also aligns with sustainable development goals. Coral powder, sourced from naturally degraded coral in oceans, offers an environmentally friendly alternative to conventional materials such as lead, which pose toxicity and environmental risks [41,42]. Harvesting practices are regulated to minimize marine ecosystem damage, with a focus on collecting dead or eroded coral. This sustainable approach ensures the dual benefit of marine conservation and resource utilization [43,44,45]. Additionally, coral’s calcium-rich composition and particle size distribution enhance its versatility in engineering materials, ensuring uniform dispersion and a consistent mechanical performance in composite matrices [46]. Trace elements such as magnesium and silica further expand its utility for specialized applications requiring unique chemical or structural characteristics [47].

Coral-derived materials show considerable promise in radiation shielding applications. Although their attenuation properties are less effective than those of lead, coral-reinforced composites provide an environmentally friendly alternative for low-to-moderate shielding needs, such as in diagnostic radiology or protective barriers. These materials offer a safer option without the health risks associated with lead-based shielding, aligning with global trends toward sustainable radiation protection technologies [48]. Additionally, evaluating other radiation shielding parameters such as atomic and electronic cross-sections and the effective atomic number (Zeff) is recommended to comprehensively assess the radiation shielding performance of coral-based composites. These parameters provide deeper insights into the interaction of materials with ionizing radiation, enabling a more detailed evaluation of their effectiveness in various shielding applications. Furthermore, incorporating uncertainty error analysis in experimental results is crucial to quantify the reliability and precision of the measurements, ensuring a robust interpretation of the mechanical and shielding data.

Despite these advantages, the study identified limitations in the material’s performance. The reduced tensile strength at higher coral filler concentrations and the heterogeneous structure observed in the 50% coral composite—marked by particle agglomeration and matrix discontinuity—pose challenges for applications requiring a high tensile and flexural performance. These findings align with similar studies on high-filler composites, which underscore the need for optimized dispersion techniques to enhance structural homogeneity and balance mechanical properties [49]. Additionally, while coral fillers improve the shielding efficiency, the attenuation remains lower than that of traditional lead-based materials, limiting their applicability in high-radiation environments. The absence of long-term durability testing is another limitation, as it precludes a comprehensive understanding of the composite’s performance under varying environmental and operational conditions.

Overall, this study highlights the broad potential use of coral-derived calcium carbonate in composite applications, emphasizing its mechanical properties, sustainability, and radiation shielding capabilities. Future research should focus on optimizing the composite formulation through hybrid filler systems, surface modifications, and advanced processing techniques to address existing limitations and further expand the material’s applicability across diverse industries.

Future studies should focus on optimizing the particle size and distribution of coral fillers within the epoxy matrix to improve tensile properties while preserving the compressive strength and radiation shielding efficiency. Investigating hybrid filler systems that combine coral-derived fillers with other sustainable materials, such as rice husk ash or seashell powder [50,51], may result in composites with enhanced multifunctional properties. Furthermore, conducting long-term durability and environmental testing is essential for evaluating the structural integrity and performance of these composites over time. Extending this research to explore the use of coral fillers in other polymer systems could further expand the potential applications of eco-friendly radiation shielding materials.

## 5. Conclusions

The prepared samples demonstrate a moderate radiation shielding performance, particularly at higher filler concentrations, effectively balancing structural reinforcement and attenuation capabilities. While the shielding efficiency is lower than conventional lead-based materials, the composites provide a sustainable, non-toxic, and environmentally friendly alternative. Compared to lead, which achieves near-complete attenuation (>99%) at similar energy levels, the coral-derived composites achieved a maximum absorption rate of 39.63% at 60 kVp, highlighting their suitability for low-to-moderate shielding applications.

This study underscores the potential use of coral-derived fillers as functional materials, particularly for applications requiring moderate radiation protection alongside enhanced compressive and flexural strength. Leveraging natural waste, these composites address the demand for eco-friendly material solutions and align with global trends in sustainable innovation. However, limitations such as a reduced tensile strength at higher filler concentrations and a lower shielding efficiency compared to lead highlight the need for further optimization.

Future research should focus on improving filler dispersion, exploring hybrid material systems, and integrating advanced processing techniques to enhance both shielding and mechanical properties. Additionally, quantitative comparisons with existing shielding materials and long-term durability testing are essential to position coral-derived composites as a viable alternative across broader industrial and medical applications.

## Figures and Tables

**Table 1:** Elemental composition of the coral powder sample analyzed by X-ray fluorescence (XRF) spectroscopy.

Elemental Oxides	Weight Percentage (%)
Calcium Oxide (CaO)	51.6
Magnesium Oxide (MgO)	1.06
Strontium oxide (SrO)	0.77
Silicon Dioxide (SiO_2_)	0.66
Sodium Oxide (Na_2_O)	0.58
Sulfur Trioxide (SO_3_)	0.4
Aluminum Oxide (Al_2_O_3_)	0.12
Chlorine (Cl)	0.09
Phosphorus Pentoxide (P_2_O_5_)	0.07
Iron Oxide (Fe_2_O_3_)	0.06
Potassium Oxide (K_2_O)	0.03

**Table 2:** Elemental composition of the pure epoxy and coral composites (Wt%).

Element	Pure Epoxy (Wt%)	Coral 25% (Wt%)	Coral 50% (Wt%)
C	81.24	52.34	46.69
O	18.55	23.18	31.79
Si	0.21	1.8	6.91
Mg	-	0.26	1.4
Al	-	0.16	2.19
S	-	2.2	-
Ca	-	7.55	8.28
Ti	-	2.81	-
Fe	-	1.13	1.61
Ba	-	8.58	-
Na	-	-	0.7
K	-	-	0.43
Total	100	100	100

**Table 3:** Comparison of radiation shielding properties between the 50% coral-derived composite and lead (0.5 mm Pb equivalent).

Material	Coral-50%	Lead
Radiation Dose (µGy)	321.5	27.75
Absorption dose (%)	24.57	93.49
Linear attenuation coefficient	0.94	5.46
Half value layer, HVL (cm)	0.74	0.13

## Data Availability

All the data are provided in the manuscript.
